# Enhanced Transdermal Delivery of Diclofenac Sodium via Conventional Liposomes, Ethosomes, and Transfersomes

**DOI:** 10.1155/2013/616810

**Published:** 2013-07-14

**Authors:** Saeed Ghanbarzadeh, Sanam Arami

**Affiliations:** ^1^Research Center for Pharmaceutical Nanotechnology, Faculty of Pharmacy, Tabriz University of Medical Sciences, Tabriz 51664-14766, Iran; ^2^Department of Pharmaceutics, Faculty of Pharmacy, Tabriz University of Medical Sciences, Tabriz 51664-14766, Iran; ^3^Student Research Committee, Faculty of Pharmacy, Tabriz University of Medical Sciences, Tabriz 51664-14766, Iran; ^4^Department of Pharmaceutical Biotechnology, Faculty of Pharmacy, Tabriz University of Medical Sciences, Tabriz 51664-14766, Iran

## Abstract

The aim of this study was to improve the transdermal permeation of Diclofenac sodium, a poorly water-soluble drug, employing conventional liposomes, ethosomes, and transfersomes. The prepared formulations had been characterized for the loaded drug amount and vesicle size. The prepared vesicular systems were incorporated into 1% Carbopol 914 gel, and a survey of *in vitro* drug release and drug retention into rat skin has been done on them using a modified Franz diffusion cell. The cumulative amount of drug permeated after 24 h, flux, and permeability coefficient were assessed. Stability studies were performed for three months. The size of vesicles ranged from 145 to 202 nm, and the encapsulation efficiency of the Diclofenac sodium was obtained between 42.61% and 51.72%. The transfersomes and ethosomes provided a significantly higher amount of cumulative permeation, steady state flux, permeability coefficient, and residual drug into skin compared to the conventional liposomes, conventional gel, or hydroethanolic solution. The *in vitro* release data of all vesicular systems were well fit into Higuchi model (RSD > 0.99). Stability tests indicated that the vesicular formulations were stable over three months. Results revealed that both ethosome and transfersome formulations can act as drug reservoir in skin and extend the pharmacologic effects of Diclofenac sodium.

## 1. Introduction

Nonsteroidal anti-inflammatory drugs (NSAIDs) are among the most frequently prescribed drugs, which are used in both acute and chronic symptoms of rheumatoid arthritis, osteoarthritis, ankylosing spondylitis, and dysmenorrhea treatment because of their analgesic, antipyretic, and anti-inflammatory roles. Their anti-inflammatory effect is due to cyclooxygenases inhibition and the consequent reduction of prostaglandin synthesis which leads to unfavorable side effects specifically on the stomach via systemic administration. Therefore, some NSAIDs are administered transdermally to achieve local or systemic effect as an alternative for oral and parenteral administration. Several formulation approaches have been developed for NSAID's transdermal administration [1–4]. The conventional pharmaceutical dosage forms which are widely administered dermally are gels, creams, and ointments. Cutaneous use of Carbopol gels is beneficial as they possess good rheological properties resulting in long remaining time at the site of administration and high drug concentration on skin. Different approaches have been performed to enhance the cutaneous passage of drugs to overcome the low skin permeability. The most frequently used approach is adding penetration enhancers into formulations. In addition, there are literatures available in which physical methods such as iontophoresis have been used for improving skin delivery of drugs [5–12]. Recently, topical delivery of drugs formulated in lipid vesicle forms had attracted considerable attention. Liposomes are microscopic spheres with an aqueous core surrounded by one or more outer shell(s) consisting of lipids in a bilayer. They are acceptable and improved carriers having ability to encapsulate both hydrophilic and lipophilic drugs. Liposomal formulations are widely used in the pharmaceutical field as drug delivery systems due to their flexibility and clinical efficacy. They have been used in drug administration via several routes such as oral, parenteral, ocular, and topical. Topical liposome formulations act as a solubilizing matrix for poorly water soluble drugs, as penetration enhancer and simultaneously a local depot which can be more effective and less toxic in comparison with conventional formulations. The liposomal gel formulations can perform better therapeutic effects than the conventional formulations, when their prolonged and controlled release property may lead to improved efficiency and better patient compliance. However, in most cases it has been shown that conventional liposomes, because of high drug deposition in the upper layers of the skin and low penetration into the deeper layers, have low efficiency as a carrier in transdermal drug delivery [13–16]. Elastic or flexible liposomes, named transfersomes, were first described by Cevc and Blume. They consist of phospholipids and a single chain surfactant such as sodium cholate, deoxycholate, Span 80 or Tween 80, which acts as an “edge activator” and destabilizes the lipid bilayers, providing greater flexibility comparing to the liposome. Transfersomes which contain up to 10% ethanol have a total lipid concentration between 5 to 10% in the final aqueous lipid suspension [7, 17–20]. Ethosomes are other novel lipid carriers which are composed of phospholipid and have high ethanol concentration (20–40%). High ethanol content of ethosomes results in being much smaller than liposomes, enhances solubility of more lipophilic drugs, and causes to be more flexible than liposomes. Besides these, disruption of intercellular lipid structure of stratum corneum by the phospholipids improves drugs permeation [21–24]. To the best of our knowledge, adding both surfactant (transfersomes) and high proportion of ethanol (ethosomes) into liposomal formulations may enable them to improve the transdermal delivery of various drugs compared to conventional liposomes. Although lipid vesicles demonstrate a promising approach for transdermal drug delivery, the practical application of these formulations on the skin has low efficiency [19, 22, 25–30]. Therefore, these vesicular systems can be incorporated into the gel formulation and applied onto the skin. In the present study, according to the aforementioned advantageous information, we produced vesicular gels of Diclofenac sodium and investigated its characteristics and *in vitro* skin permeation.

## 2. Materials and Methods

### 2.1. Materials

Diclofenac sodium (CAS 15307-79-6) was supplied by Alborz Company (Ghazvin, Iran). Disodium phosphate, monopotassium phosphate, ethanol, Carbopol 914, soya lecithin, cholesterol, and Span 80 were obtained from Merck Company (Darmstadt, Germany).

### 2.2. Preparation of Reference Formulations

Several solvent systems have been developed to increase the solubility of active ingredients. These solvents must incorporate with substances having different lipophilicity degrees. In this study, reference hydroethanolic formulation was prepared at laboratory scale and at room temperature by dissolving Diclofenac sodium (1% w/w) in an ethanol : water (20 : 80) mixture. The appropriate quantity of Carbopol 914 powder was dispersed into hydroethanolic solution containing Diclofenac sodium (1% w/w) under constant stirring with magnetic stirrer and allowed to hydrate for 24 h at room temperature to swell. The dispersion was neutralized using triethanolamine (0.5% w/w).

### 2.3. Preparation of Conventional Liposomes, Ethosomes, and Transfersomes

Conventional liposomes are composed of phospholipid and cholesterol. The most common phospholipid is phosphatidylcholine obtained from soybean or egg yolk. In the present study, liposomes were prepared by a modified ethanol injection method. Briefly, phosphatidylcholine, cholesterol, and drug were dissolved in ethanol and injected slowly into the aqueous medium under mixing by homogenizer [31, 32]. 

As well as conventional liposomes, a range of structurally similar vesicles have been developed, including ethosomes and transfersomes.

Similar to liposomes, ethosomes are composed of phospholipids but can contain 20–40% ethanol. Ethosomes were prepared by first dissolving the lipids and drug in ethanol, then adding the aqueous component slowly as a fine stream under mixing by homogenizer for 60 min [33–36]. The elastic liposomes were prepared by rotary evaporation method using Span 80 as surfactant, phospholipid, cholesterol, and drug. The accurately weighed amounts of phospholipid, surfactant, cholesterol, and drug were taken in a clean, dry, round-bottom flask, and this lipid mixture was dissolved in small quantity of chloroform-methanol mixture (3 : 1). The organic solvent was removed by rotary evaporation under reduced pressure at 45°C. The deposited lipid film was hydrated with drug containing aqueous solution by rotation for 1 hr [19, 20, 37, 38]. Subsequently, processing by filter extrusion method, small unilamellar vesicles were produced.

### 2.4. Preparation of Vesicular Gel

Topical vesicular gel formulations were prepared incorporating vesicular dispersions containing drug (separated from the unentrapped drug) into the Carbopol gel under mechanical stirring. 

A fixed concentration of Diclofenac sodium was used in all formulations to make the vehicles effect on percutaneous absorption comparable.

### 2.5. Characterization of Vesicles

#### 2.5.1. Size Distribution

Mean particle size of vesicles was determined by photon correlation spectroscopy using Shimadzu particle size analyzer model SALD 2101 (Japan). Diluted liposome suspension was added to the sample dispersion unit while stirring at room temperature (in order to reduce the inter particle aggregation). The assay has been performed in triplicate.

#### 2.5.2. Encapsulation Efficiency Determination

The liposome-encapsulated Diclofenac sodium was separated from unentrapped drug by dialysis method [31, 32]. Liposomes were lysed with ethanol, and the released Diclofenac sodium was assayed spectrophotometrically at 276 nm. The percent of encapsulation efficiency (EE%) was then calculated according to the following equation:

(1) $$\begin{array}{c} \text{EE}\%=\frac{\text{drug}\;\text{content}}{\text{total}\;\text{drug}\;\text{added}}\times 100. \end{array}$$

Each experiment has been done in triplicate, and the data reported is the mean value.

#### 2.5.3. Zeta Potential Determination

Surface charge of drug-loaded vesicles was determined using Zetasizer (Malvern Instruments, Malvern, UK). Analysis time was kept 60 sec, and average zeta potential of the vesicles was determined.

### 2.6. Physical Stability Studies

Physical stability tests of the prepared vesicles were carried out to investigate the aggregation of vesicles and leakage of drug from them during storage. The prepared Diclofenac sodium vesicles were stored in transparent vials covered with plastic cap at ambient temperature and 4°C for three months. The physical stability was evaluated by mean vesicle size, EE%, and zeta potential measurement over a three-month period. Samples from each vesicle were withdrawn monthly, and the particle size, EE%, and zeta potential of the vesicles were measured as described previously.

### 2.7. Skin Permeation and Drug Deposition Studies

Hairless rat skin was used for *in vitro* transdermal permeation studies. Rats were sacrificed using excessive diethyl ether as anesthesia. Full thickness (1.2–1.4 mm) ventral skin of male Wistar rats (160 ± 25 g) was excised and shaved with an electric shaver. Any extraneous subcutaneous fat was removed from the dermal surface, and the skin was mounted between the donor and receptor chamber of modified Franz diffusion cell (the surface area available for diffusion was 2.51 cm^2^, and receptor volume was 26 cm^3^) containing isotonic phosphate buffered saline (PBS) (pH 7.4). Skins were kept in PBS for 6 h to become saturated before they were used for permeation studies. Accurately weighed different formulations (amount equivalent to 200 mg of drug) were applied on the skin surface and spread by means of a spatula ensuring that there were no air bubbles between the formulation and donor surface. The receptor fluid was maintained at 37 ± 0.5°C and continuously stirred at 100 rpm using a magnetic stirrer. Receptor medium (2 mL) was withdrawn at specific time intervals over a 24 h period and immediately replaced with fresh prewarmed buffer. The samples were quantified using a validated HPLC method [11]. Each formulation was investigated in 3 separate cells.

This study was conducted in accordance with the Guide lines of the Care and Use of Laboratory Animals of Tabriz University of Medical Sciences, Tabriz-Iran (National Institutes of Health Publication no. 85-23, revised 1985).

### 2.8. Determination of the Amount of Remained Drug in the Skin

At the end of the permeation experiments, the skin was carefully removed from the Franz cell and the remaining formulation on the skin surface was swabbed and washed first with PBS pH 7.4 and then with methanol. The procedure was repeated twice to ensure that no traces of formulation were left onto skin surface. The permeation area of the skin was excised, weighed, and then cut into small pieces to extract the drug content present in skin with ethanol. The resulting solutions were centrifuged (1500 rpm), and the Diclofenac sodium levels were measured and expressed as percent of initially applied drug. 

### 2.9. Calculation of Skin Permeation Parameters

The cumulative amount of drug permeated per unit area was plotted as a function of time. The flux was calculated from the slope of the linear portion. The permeability coefficient (*K*_*p*_) of Diclofenac sodium across rat skin was calculated using relation derived from Fick's first law of diffusion, which is expressed by the following equation:

(2) $$\begin{array}{c} K_{p}=\frac{J}{C}, \end{array}$$

where *J* is the flux and *C* is the drug concentration in donor compartment. 

### 2.10. Statistical Analysis

Data analysis was carried out using Microsoft Excel 2010. Results are expressed as mean ± standard deviation (*n* = 3). Statistically significant differences were determined using the analysis of variance (ANOVA) with *P* < 0.05 as a minimal level of significance.

## 3. Results

### 3.1. Characterization of Liposomes

The mean size of prepared conventional liposomes and ethosomes was 152 ± 21.3 nm and 202 ± 20.6 nm, respectively, with low homogeneity (0.35 polydispersity). However, the mean size of the transfersomes was in the range of 426 ± 32.6 nm which was significantly reduced to 145 ± 6.6 nm with high homogeneity (0.1 polydispersity) after extraction. The mean encapsulation efficiency of the drug in the prepared conventional liposomes, ethosomes, and transfersomes were 42.61 ± 3.62%, 51.72 ± 4.36%, and 46.73 ± 5.21%, respectively.

### 3.2. Physical Stability Study

Physical stability study of the prepared vesicles showed higher percentage of drug retained in formulations stored at refrigerated than at room temperature after three months. This may be due to higher fluidity of lipid bilayers at higher temperatures resulting in higher drug leakage. Analysis of drug leakage study data revealed that vesicular gel was significantly more stable than vesicles suspension, and also both types of formulations were significantly more stable at refrigerated temperature than at room temperature (Table 1). Vesicles also were found to be reasonably stable in terms of aggregation and fusion (Table 2). In accordance with the results, it can be concluded that at room temperature and 4°C, there was slightly but insignificantly increase in the particle size. The stability of vesicles improved after incorporation into gel maybe due to prevention of fusion of vesicles. Results suggest that keeping the vesicular product in refrigerated conditions minimizes the unstability problems of vesicles.

### 3.3. *In Vitro* Permeation Studies

**Figure 1:**
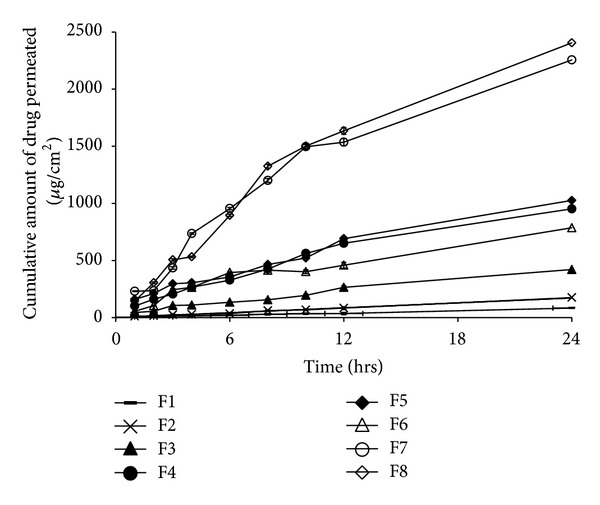
*In vitro* drug penetration from the different formulations (Table 1) through rat skin in Franz diffusion cell at 37°C ± 0.5°C.

Equal amounts of Diclofenac sodium from different formulations, including solution, dispersion, and Carbopol gel incorporated form, were applied on the skin surface in donor compartment to make a comparison among their drug penetration ability through rat skin. Amount of Diclofenac sodium permeated through excised mouse skin over 24 h was plotted versus time.  Figure 1 represents the penetration profile of Diclofenac sodium (penetrated amount of Diclofenac sodium/cm^2^ skin surface versus time) through excised rat skin from different prepared formulations.

The mechanism of release kinetics was evaluated by fitting the permeation data to the zero-order and Higuchi diffusion models. All permeation profiles of vesicular dispersions and gels fit well into the Higuchi diffusion model (RSD > 0.99), and a linear relationship was found between the amount of drug released and the square root of time. It could be concluded that the vesicles acted as reservoir systems for continuous delivery of the encapsulated drug.

Table 3 presents the permeation parameters of Diclofenac sodium through excised rat skin and accumulated amount of remained and permeated drug over 24 h from different formulations.

It is clear that the cumulative amount of drug permeated across rat skin after 24 h for both hydroethanolic solution and Carbopol gel (84.3 ± 2.3 and 176.6 ± 6 *μ*g/cm^2^) was significantly lower in comparison with vesicular systems, specially ethosomal or transfersomal formulations (2256.9 ± 68.3 and 2405.5 ± 110.6), indicating that vesicular systems can improve skin delivery of hydrophobic drugs such as Diclofenac sodium. On the other hand, the cumulative amount of drug permeated from liposomal formulations after 24 h was significantly less than that drug permeated from the ethosomal or transfersomal formulations (*P* < 0.05). Similar conclusions could be obtained upon analysis of the drug flux and permeability coefficient of the same formulations. The mean steady state flux and permeability coefficient ranged from 3.36 ± 0.68 (hydroethanolic solution) to 100.57 ± 3.61 (transfersomal gel) *μ*g/cm^2^/h and 0.336 ± 0.06 (hydroethanolic solution) to 10.05 ± 0.36 (transfersomal gel) cm/h, respectively. Results indicated that the flux and permeability coefficient of ethosomes or transfersomes were 15- and 30-fold higher than Carbopol gel and hydroethanolic solution of drug, respectively. Results also revealed that, compared to vesicular dispersions, incorporation of vesicular formulations into gel preparation could significantly increase flux and *K*_*p*_ up to 3 times (*P* < 0.05). It can be concluded from the results that ethosomes or transfersomes in both vesicular dispersion and vesicular gel forms could penetrate and deposit Diclofenac sodium 2-3 times more than liposomes. High deposition percent indicated that ethosomes and transfersomes could provide a drug reservoir in skin to prolong the effect of Diclofenac sodium at the interval times between administrations.

## 4. Discussion

Liposomes as bimolecular phospholipid bilayers are capable of encapsulating hydrophobic, hydrophilic, and amphiphilic drugs. Liposomes by diffusing into the stratum corneum, disrupting the bilayer fluidity in the stratum corneum, loosening the lipid structure of the stratum corneum, and providing impaired barrier function of these layers to the drug act as penetration enhancers. Moreover, some studies reported that phospholipids in liposomes may mix with the stratum corneum lipids creating a lipid-enriched environment. This lipid enriched layer in the skin is preferred by lipophilic drugs, resulting in enhanced skin uptake. In some cases, phospholipids themselves can increase the solubility of lipophilic drugs such as Diclofenac sodium [13, 39–41]. Many studies have reported increased *in vitro* skin permeation of ethosomal formulations. Ethosomes not only like classical liposomes contain phospholipids but also contain high levels of ethanol (up to 45%) and are able to enhance the penetration to deep tissues and the systemic circulation. Although the exact method of action of ethosomes remains unclear, ethanol is a well-known permeation enhancer, and mechanisms of these carriers in improving permeation are explained by their alcohol content as penetration enhancers. Ethanol interacts with lipid molecules in the polar head group region, resulting in a reduced phase transition temperature (*T*_*m*_) of the stratum corneum lipids and increased fluidity which leads to improved membrane permeability. Ethanol may also provide the vesicles with softness and flexibility that allows ethosomes to squeeze more easily and penetrate into the deeper layers of the skin due to their increased alcohol component. Ethosomal preparations were found to be much more effective permeation enhancers than hydroethanolic solution, ethanol, or an ethanolic phospholipid solution, indicating that permeation enhancement from ethosomes was much greater than would be expected from ethanol alone [22, 29, 33, 35, 36, 42–44]. 

Transfersomes are vesicles composed of phospholipids, with 10–25% surfactant and 3–10% ethanol. Transfersomes up to 500 nm can squeeze to penetrate the stratum corneum barrier spontaneously. It has been demonstrated that as transfersomes due to their elasticity and high deformable structure could reach deeper dermal tissues and even the systemic circulation, they ensure higher skin permeation than conventional liposomes. When drugs remain strongly associated with the vesicles, elastic vesicles can be used to transfer drugs rapidly into the deeper layers of the stratum corneum, and subsequently administered drugs can permeate into the viable epidermis. Therefore, elastic vesicles have superior characteristics compared to rigid conventional vesicles [19, 22, 37].

## 5. Conclusions

The comparative evaluation of *in vitro* skin permeability toward several vesicular preparations containing Diclofenac sodium is considered to be useful in preformulation step to predict the best formulation in the development of topical formulations with higher drug penetration through rat skin. According to our results, vesicular systems, especially when incorporated into gel preparation, had high permeability, and among the vesicular systems, ethosomes and transfersomes had better influence in Diclofenac sodium permeation through rat skin.

## Figures and Tables

**Table 1:** Composition, particle size, polydispersity index (PDI), zeta potential, and encapsulation efficiency percent (EE%) of prepared vesicles.

Formulation	Phospholipid (mg/mL)	Cholesterol (mg/mL)	Span 80 (mg/mL)	Particle size (nm)	PDI	Zeta potential (mV)	EE%
Liposome	100	30	—	152 ± 21.3	0.37	−39.2 ± 3.9	42.61 ± 3.62
Ethosome	100	30	—	202 ± 20.6	0.34	−41.2 ± 2.1	51.72 ± 4.36
Transfersome	100	30	30	145 ± 6.6	0.11	−38.9 ± 4.1	46.73 ± 5.21

**Table 2:** Stability of prepared vesicles during storage at 4°C and 25°C for three months.

Formulation (code)	Particle size (nm)		Zeta potential (mV)		EE%	
	Initial	After 3 months	Initial	After 3 months	Initial	After 3 months
Liposomal dispersion (F3)						
4°C	152 ± 21.3	193 ± 23.6	−39.2 ± 3.9	−40.2 ± 2.1	42.61 ± 3.62	36.2 ± 1.9
25°C	152 ± 21.3	219 ± 16.4	−39.2 ± 3.9	−36.1 ± 0.9	42.61 ± 3.62	32.1 ± 2.6
Ethosomal dispersion (F4)						
4°C	202 ± 20.6	235 ± 30.1	−41.2 ± 2.1	−42.3 ± 1.2	51.72 ± 4.36	44.6 ± 1.8
25°C	202 ± 20.6	266 ± 8.6	−41.2 ± 2.1	−39.2 ± 3.1	51.72 ± 4.36	39.9 ± 3.2
Transfersomal dispersion (F5)						
4°C	145 ± 6.6	189 ± 9.5	−38.9 ± 4.1	−36.2 ± 1.1	46.73 ± 5.21	42.3 ± 0.9
25°C	145 ± 6.6	223 ± 19.2	−38.9 ± 4.1	−39.2 ± 0.8	46.73 ± 5.21	35.2 ± 1.1
Liposomal gel (F6)						
4°C	152 ± 21.3	169 ± 10.2	−39.2 ± 3.9	−39.9 ± 3.2	42.61 ± 3.62	39.5 ± 1.0
25°C	152 ± 21.3	182 ± 12.0	−39.2 ± 3.9	−37.2 ± 2.5	42.61 ± 3.62	38.2 ± 2.6
Ethosomal gel (F7)						
4°C	202 ± 20.6	221 ± 16.2	−41.2 ± 2.1	−40.2 ± 1.9	51.72 ± 4.36	46.8 ± 2.4
25°C	202 ± 20.6	230 ± 20.0	−41.2 ± 2.1	−41.1 ± 1.2	51.72 ± 4.36	44.1 ± 1.7
Transfersomal gel (F8)						
4°C	145 ± 6.6	186 ± 9.2	−38.9 ± 4.1	−35.2 ± 2.9	46.73 ± 5.21	43.6 ± 2.6
25°C	145 ± 6.6	196 ± 11.1	−38.9 ± 4.1	−38.9 ± 3.1	46.73 ± 5.21	40.3 ± 1.1

**Table 3:** Permeated amount of Diclofenac sodium at 24 h, flux, permeability coefficient, and residual drug remaining in the skin as percent of initially applied drug for the formulations. Results are revealed as mean ± standard deviation (*n* = 3).

Formulation (code)	Permeated amount at 24 h (*µ*g/cm^2^)	Flux (*µ*g/cm^2^/h)	Permeability coefficient (*K*_*p*_) × 10^−3^ (cm/h)	Residual drug (%)
Hydroethanolic solution (F1)	84.3 ± 2.3	3.36 ± 0.68	0.336 ± 0.06	0.89 ± 0.26
Carbopol gel (F2)	176.6 ± 6.5	7.17 ± 1.69	0.717 ± 0.16	2.32 ± 0.95
Liposomal dispersion (F3)	421.2 ± 35.6	16.32 ± 2.35	1.632 ± 0.23	4.32 ± 1.12
Ethosomal dispersion (F4)	952.6 ± 95.3	37.77 ± 6.2	3.777 ± 0.6	10.58 ± 2.01
Transfersomal dispersion (F5)	1026.6 ± 63.8	37.87 ± 3.15	3.781 ± 0.32	12.26 ± 3.25
Liposomal gel (F6)	786.0 ± 86.3	29.13 ± 5.24	2.913 ± 0.52	12.62 ± 2.68
Ethosomal gel (F7)	2256.9 ± 68.3	91.69 ± 6.51	9.169 ± 0.65	23.9 ± 1.86
Transfersomal gel (F8)	2405.5 ± 110.6	100.57 ± 3.61	10.057 ± 0.36	21.32 ± 2.63
